# Supplementary material for: Hybrid Models and Biological Model Reduction with PyDSTool
Source: PLoS Comput Biol. 2012 Aug 9;8(8):e1002628. doi: 10.1371/journal.pcbi.1002628 (PMC3415397; doi:10.1371/journal.pcbi.1002628)
Supplement: Text S4 — Complete source code for the PyDSTool package (version 0.88.120504). Includes API documentation and help files linking to web pages. This file is identical to the current public release on Sourceforge.net. (ZIP) [file pcbi.1002628.s004.zip › PyDSTool/html/PyDSTool.Toolbox.PRCtools-pysrc.html]

xml version="1.0" encoding="ascii"?


PyDSTool.Toolbox.PRCtools


| Home | Trees | Indices | Help | PyDSTool |
| --- | --- | --- | --- | --- |

Package PyDSTool ::
Package Toolbox ::
Module PRCtools

[hide private]

[frames] | no frames]

# Source Code for Module PyDSTool.Toolbox.PRCtools

```
  1  """Toolbox for phase response curves measured by finite perturbations 
  2  """ 
  3   
  4  import numpy as np 
  5  from PyDSTool import Pointset, Model, embed 
  6  from PyDSTool.Trajectory import pointset_to_traj 
  7   
  8  from pylab import figure, plot 
  9   
 10  #__all__ = [] 
 11   
 12  # ------------------------------------------------- 
 13   


14 -def one_period_traj(model, ev_name, ev_t_tol, ev_norm_tol, T_est, 
 15                      verbose=False, initial_settle=6, restore_old_ics=False, 
 16                      use_quadratic_interp=False):


17      """ 
 18      Utility to extract a single period of a limit cycle of the model using forward 
 19      integration, up to a tolerance given in terms of both the period and the norm of the 
 20      vector of variables in the limit cycle at the period endpoints. 
 21   
 22      Requires a non-terminal event in the model that is detected exactly once per period. 
 23      Assumes model initial conditions are already in domain of attraction for limit cycle. 
 24   
 25      T_est is an initial estimate of period. 
 26      use_quadratic_interp option (default False) indicates whether to make the returned 
 27      trajectory interpolated more accurately using quadratic functions rather than linear ones. 
 28      This option takes a lot longer to complete! 
 29   
 30      The model argument can be an instance of a Generator class or Model class. 
 31   
 32      Returned trajectory will have name 'one_period'. 
 33      """ 
 34      if not isinstance(model, Model.Model): 
 35          # temporarily embed into a model object 
 36          model = embed(model) 
 37      if use_quadratic_interp: 
 38          old_interp_setting = model.query('algparams')['poly_interp'] 
 39          model.set(algparams={'poly_interp': True}) 
 40      trajname = '_test_period_' 
 41      old_ics = model.query('ics') 
 42      settle = initial_settle 
 43      tries = 1 
 44      success = False 
 45      while not success and tries < 8: 
 46          model.compute(trajname=trajname, tdata=[0,T_est*(settle+0.2)], force=True) 
 47          evts = model.getTrajEventTimes(trajname, ev_name) 
 48          all_evs = model.getTrajEventTimes(trajname) 
 49          if len(evts) <= 2: 
 50              raise RuntimeError("Not enough events found") 
 51          ref_ic = model(trajname, evts[-1]) 
 52          t_check = 10000*np.ones((tries,),float) 
 53          norm_check = 10000*np.ones((tries,),float) 
 54          T = np.zeros((tries,),float) 
 55          look_range = range(1, min((tries+1, len(evts)))) 
 56          if verbose: 
 57              print "\n Tries: ", tries, "\n" 
 58          for lookback in look_range: 
 59              try: 
 60                  d_evts = [evts[i]-evts[i-lookback] for i in \ 
 61                                      range(lookback, len(evts))] 
 62              except KeyError: 
 63                  # no more events left to look back at 
 64                  break 
 65              else: 
 66                  prev_val = model(trajname, evts[-(1+lookback)]) 
 67                  t_check[lookback-1] = abs(d_evts[-1]-d_evts[-2]) 
 68                  norm_check[lookback-1] = np.linalg.norm(ref_ic-prev_val) 
 69                  T[lookback-1] = d_evts[-1] 
 70          T_est = T[0] 
 71          t_ix = np.argmin(t_check) 
 72          n_ix = np.argmin(norm_check) 
 73          ix1 = min((t_ix, n_ix)) 
 74          ix2 = max((t_ix, n_ix)) 
 75          if verbose: 
 76              print t_check, norm_check, T 
 77              print ix1, ix2 
 78          if t_check[ix1] < ev_t_tol and norm_check[ix1] < ev_norm_tol: 
 79              success = True 
 80              T_final = T[ix1] 
 81          elif ix1 != ix2 and t_check[ix2] < ev_t_tol and norm_check[ix2] < ev_norm_tol: 
 82              success = True 
 83              T_final = T[ix2] 
 84          else: 
 85              tries += 1 
 86              settle = tries*2 
 87              model.set(ics = ref_ic) 
 88      if success: 
 89          model.set(ics=ref_ic, tdata=[0, T_final]) 
 90          model.compute(trajname='one_period', force=True) 
 91          ref_traj = model['one_period'] 
 92          # insert the ON event at beginning of traj 
 93          ref_traj.events[ev_name] = Pointset(indepvararray=[0], 
 94                                          coordarray=np.array([ref_ic.coordarray]).T, 
 95                                          coordnames=ref_ic.coordnames) 
 96          ref_pts = ref_traj.sample() 
 97          # restore old ICs 
 98          if restore_old_ics: 
 99              model.set(ics=old_ics) 
100          if use_quadratic_interp: 
101              model.set(algparams={'poly_interp': old_interp_setting}) 
102          return ref_traj, ref_pts, T_final 
103      else: 
104          raise RuntimeError("Failure to converge after 80 iterations")

105   
106   


107 -def _default_pert(model, ic, pertcoord, pertsize):


108      ic[pertcoord] += pertsize 
109      return ic

110   
111   


112 -def finitePRC(model, ref_traj_period, evname, pertcoord, pertsize=0.05, 
113                settle=5, verbose=False, skip=1, do_pert=_default_pert, 
114                keep_trajs=False, stop_at_t=np.inf, force_T=np.nan):


115      """Pass a Generator or Model instance for model. 
116      Pass a Trajectory or Pointset for the ref_traj_period argument. 
117      Pass the event name in the model that indicates the periodicity. 
118      Use skip > 1 to sub-sample the points computed along the trajectory at 
119       the skip rate. 
120      Use a do_pert function to do any non-standard perturbation, e.g. if there 
121       are domain boundary conditions that need special treatment. This function 
122       takes four arguments (model, ic, pertcoord, pertsize) and returns the new 
123       point ic (not just ic[pertcoord]). 
124      Use settle=0 to perform no forward integration before the time window in 
125       which the perturbation will be applied (e.g. for non-cycles). 
126      Use stop_at_t to calculate a partial PRC, from perturbation time 0 to this 
127       value. 
128      Use force_T to force the period to be whatever value you like. 
129   
130      Note: Depending on your model, there may be regions of the PRC that are 
131      offset by a constant amount to the rest of the PRC. This is a "wart" that 
132      needs improvement. 
133      """ 
134      if not isinstance(model, Model.Model): 
135          # temporarily embed into a model object 
136          model = embed(model) 
137      try: 
138          all_pts = ref_traj_period.sample() 
139          ref_pts = all_pts[::skip] 
140          if ref_pts[-1] != all_pts[-1]: 
141              # ensure last point at t=T is present 
142              ref_pts.append(all_pts[[-1]]) 
143          if np.isnan(force_T): 
144              T = ref_traj_period.indepdomain[1]-ref_traj_period.indepdomain[0] 
145          else: 
146              T = force_T 
147      except AttributeError: 
148          # already passed points 
149          ref_pts = ref_traj_period[::skip] 
150          if ref_pts[-1] != ref_traj_period[-1]: 
151              ref_pts.append(ref_traj_period[[-1]]) 
152          if np.isnan(force_T): 
153              T = ref_traj_period.indepvararray[-1]-ref_traj_period.indepvararray[0] 
154          else: 
155              T = force_T 
156      ref_ts = ref_pts.indepvararray 
157      PRCvals = [] 
158      t_off = 0 
159      if verbose: 
160          print "Period T =", T 
161      for i, t0 in enumerate(ref_ts): 
162          if t0 > stop_at_t: 
163              break 
164          ic = ref_pts[i] 
165          ic = do_pert(model, ic, pertcoord, pertsize) 
166          if verbose: 
167              print i, "of", len(ref_ts), ": t0 = ", t0, "of", T, "  t_end", settle*T+t0 
168              print "   ", ic 
169          model.set(ics=ic.copy(), tdata=[0, (settle+1)*T+t0]) 
170          if keep_trajs: 
171              model.compute(trajname='pert_%i'%i, force=True) 
172              evts = model.getTrajEventTimes('pert_%i'%i, evname) 
173          else: 
174              model.compute(trajname='pert', force=True) 
175              evts = model.getTrajEventTimes('pert', evname) 
176          if verbose: 
177              print "    Last event:", evts[-1] 
178          val = -np.mod(evts[-1]+t0, T)/T 
179          if abs(val) > 0.5: 
180              val = val+1 
181          PRCvals.append(val) 
182      return Pointset(coordarray=[PRCvals], coordnames=['D_phase'], indepvararray=ref_ts[:i], indepvarname='t')

183   
184   


185 -def compare_pert(model, ref_traj_period, evname, pertcoord, pertsize, t0, settle=5, 
186                   do_pert=_default_pert, fignum=None):


187      """Show perturbed and un-perturbed trajectories starting at t0, with given perturbation function 
188      do_pert. 
189      """ 
190      if not isinstance(model, Model.Model): 
191          # temporarily embed into a model object 
192          model = embed(model) 
193      try: 
194          all_pts = ref_traj_period.sample() 
195      except AttributeError: 
196          raise TypeError("Must pass a reference trajectory object") 
197      else: 
198          T = ref_traj_period.indepdomain[1]-ref_traj_period.indepdomain[0] 
199      ref_ts = all_pts.indepvararray 
200   
201      assert t0 > ref_ts[0] and t0 < ref_ts[-1], "t0 out of range" 
202      ic = do_pert(model, ref_traj_period(t0), pertcoord, pertsize) 
203      model.set(ics=ic, tdata=[0,settle*T+t0]) 
204      model.compute(trajname='compare_pert', force=True) 
205      evts = model.getTrajEventTimes('compare_pert', evname) 
206      PRC_val = -np.mod(evts[-1]+t0, T)/T 
207      if abs(PRC_val) > 0.5: 
208          PRC_val += 1 
209      print "t0 = %.6f, PRC value = %f" % (t0, PRC_val) 
210   
211      if fignum is not None: 
212          figure(fignum) 
213      plot(all_pts['t'], all_pts[pertcoord], 'g') 
214      while all_pts.indepvararray[-1] < settle*T+t0: 
215          # plot additional periodic cycles to compare with pert traj 
216          all_pts.indepvararray += T 
217          plot(all_pts['t'], all_pts[pertcoord], 'g') 
218      pert_pts = model.sample('compare_pert') 
219      pert_pts.indepvararray += t0 
220      plot(pert_pts['t'], pert_pts[pertcoord], 'r') 
221      return pert_pts

222   
223   


224 -def fix_PRC(PRC, tol=0.01):


225      """Experimental and hopefully unnecessary utility to 'fix' PRC data with phase jumps 
226      of 1/2 or a whole cycle due to computational problems.""" 
227      new_vals = [] 
228      for ix, phase in enumerate(PRC['D_phase']): 
229          if phase > 0.5-tol: 
230              phase = phase - 0.5 
231          elif phase < tol-0.5: 
232              phase = phase + 0.5 
233          else: 
234              continue 
235          # drop through 
236          new_vals.append( (ix, phase) ) 
237      for ix, phase in new_vals: 
238          PRC.coordarray[0][ix] = phase 
239      return PRC

240
```

  


| Home | Trees | Indices | Help | PyDSTool |
| --- | --- | --- | --- | --- |

|  |  |
| --- | --- |
| Generated by Epydoc 3.0.1 on Fri May 4 15:24:16 2012 | http://epydoc.sourceforge.net |
